# Cytoreductive prostate radiotherapy in oligometastatic prostate cancer: a single centre analysis of toxicity and clinical outcome

**DOI:** 10.3332/ecancer.2017.786

**Published:** 2017-11-30

**Authors:** Giulia Riva, Giulia Marvaso, Matteo Augugliaro, Dario Zerini, Cristiana Fodor, Gennaro Musi, Ottavio De Cobelli, Roberto Orecchia, Barbara Alicja Jereczek-Fossa

**Affiliations:** 1 Department of Radiotherapy, European Institute of Oncology, 20141 Milan, Italy; 2 Department of Oncology and Hemato-Oncology, University of Milan, 20122 Milan, Italy; 3 Department of Urology, European Institute of Oncology, 20141 Milan, Italy; 4 Scientific Direction, European Institute of Oncology, 20141 Milan, Italy

**Keywords:** oligometastases, prostate cancer, oligometastatic prostate cancer, radiotherapy

## Abstract

**Objectives:**

The current standard of care for patients with metastatic prostate cancer (mPCa) at diagnosis is androgen deprivation therapy (ADT) with or without anti-androgen and chemotherapy. The aim of this study was to define the role of a local radiotherapy (RT) treatment in the mPCa setting.

**Methods:**

We retrospectively reviewed data of patients with PCa and bone oligometastases at diagnosis treated in our institution with ADT followed by cytoreductive prostate-RT with or without RT on metastases. Biochemical and clinical failure (BF, CF), overall survival (OS) and RT-toxicity were assessed.

**Results:**

We identified 22 patients treated with ADT and external-beam RT on primary between June 2008 and March 2016. All of them but four were also treated for bone metastases. RT on primary with moderately and extremely hypofractionated regimes started after 10.3 months (3.9–51.7) from ADT. After a median follow-up of 26.4 months (10.3–55.5), 20 patients are alive. Twelve patients showed BF after a median time of 23 months (14.5–104) and CF after a median of 23.6 months (15.3–106.1) from the start of ADT. Three patients became castration resistant, starting a new therapy; median time to castration resistance was 31.03 months (range: 29.9–31.5 months). According to the Radiation Therapy Oncology Group/European Organization for Research and Treatment of Cancer (RTOG/EORTC), only one patient developed acute grade 3 genitourinary toxicity. No late grade >2 adverse events were observed.

**Conclusion:**

Prostate RT in oligometastatic patients is safe and offers long-lasting local control. When compared to ADT alone, RT on primary seems to improve biochemical control and long-term survival; however, this hypothesis should be investigated in prospective studies. Further research is warranted.

## Introduction

Oligometastatic prostate cancer (PCa), defined in five or fewer metastatic sites, is a relatively new concept [1].

Since the introduction of anatomical and functional imaging with multiparametric magnetic resonance imaging (MRI) and choline or novel nuclear tracer, such as prostate-specific membrane antigen (PSMA) positron emission tomography–computed tomography (PET–CT), together with early prostate-specific antigen (PSA) detection, the rate of patients with synchronous metastases at diagnosis has increased, reaching at least 5% of all newly diagnosed cancers [2].

Androgen deprivation therapy (ADT) remains the standard of care (SOC) with a five-year relative survival of 28%, compared to the 100% of patients with organ-confined or locally advanced PCa [3].

Recently, the E3805 (CHARTEED) and Systemic Therapy in Advancing or Metastatic Prostate Cancer: Evaluation of Drug Efficacy (STAMPEDE) studies have reported that adding docetaxel improves overall survival (OS) in men who are fit for chemotherapy with newly diagnosed metastatic PCa (mPCa) commencing ADT [4, 5]. Moreover, although generally modest, a landscape of currently available therapies for castration-resistant disease (such as cabazitaxel, abiraterone acetate, enzalutamide and radium-223) provided some benefits in terms of OS [6].

Considering that local treatment as prostatectomy and radiation therapy were reserved only for patients with organ-confined or locally advanced disease, treatment of oligometastatic disease was quite limited. The scenario has changed in the past decade, based on the fact that oligometastatic PCa could be recognised as a clinically significant state separate from the polymetastatic disease and considered to be less aggressive than other metastatic phenotypes [7, 8]. Not all patients with mPCa share the same prognosis. Selected individuals with oligometastatic PCa might benefit from local therapies.

Recent clinical studies support an oncologic role for surgery or radiotherapy (RT) in mPCa [9, 10]. Local treatment is also supported by biological basis so as to eliminate the source of tumour-promoting factors, destroy the origin of metastatic cells and stop the self-seeding process.

The aim of this study is to retrospectively report and evaluate the possible role of RT of primary tumour associated with ADT in patients affected by oligometastatic PCa at the time of diagnosis, in order to avoid local progression, retard time to castration resistance and prolong survival.

## Materials and methods

### Study population

We retrospectively reviewed the data of patients based on the following inclusion criteria:

- histological diagnosis of PCa;
- documented stage IV (M1b) disease at the time of diagnosis based on the American Joint Committee on Cancer (AJCC) tumour, node and metastases (TNM) stage;
- number of bone metastases less or equal to five (based on the current definition of oligometastatic disease) [11];
- good performance status (Eastern Cooperative Oncology Group ECOG 0-1) at the time of RT;
- external-beam radiation therapy (EBRT) as local cytoreductive treatment;
- neoadjuvant ADT and no progression of cancer before starting RT;
- written informed consent for RT;
- written informed consent for use of anonymised data for research and educational purposes.

Radiological examinations with Choline C-11 PET (Choline C-11 PET), whole body CT, whole body MRI and/or bone scan findings were used to confirm diagnosis of oligometastatic disease and clinical progression.

The study was a part of general stereotactic body radiation therapy (SBRT) and image-guided RT (IGRT) PCa research notified to the Ethical Committee of the European Institute of Oncology, Milan, Italy (notifications No. 79/10, 86/11, 87/11 and 93/11). All patients gave written informed consent for treatment and written informed consent for use of the anonymised data for research and educational purposes.

### Radiotherapy treatment

Starting RT treatment of the primary tumour was discussed in our multidisciplinary tumour board, taken into account after a good clinical and radiological response from an initial treatment with ADT for each patient. Prostate-directed irradiation aim was proposed in order to postpone PCa progression and defer other treatments, specifically chemotherapy.

Patients underwent RT with moderately hypofractionated and extremely hypofractionated regimes using image guided intensity modulated RT(IG-IMRT) [12].

Among our patients, 17 were treated on prostate and seminal vesicles with IG-Static Step and Shoot IMRT by Vero (BrainLab, D/MHI, Japan) with seven fields of 6-MV photons and three with IG-Volumetric Modulated Arc Therapy (RapidArc, Varian Medical Systems) with seven fields of 6-MV photons. Pelvic lymph nodes irradiation was performed only in one case of documented stage cN1.

RT dose to the prostate was at least 7 Gy per fraction to a median biological effective dose (BED) of 173 Gy, using an a/b of 1.5. Primary tumour was irradiated with the following BEDs: 90–110 Gy (two patients), 111–150 Gy (six patients), 151–180 Gy (seven patients) and >180 Gy (five patients) [13].

Treatment included irradiation of one or more bone metastases sites for 16 patients: for 12 patients, the RT cycle was synchronous including prostate and bone at the same time, while for four patients, RT of the primary was performed after a median of 11.6 months (range: 2.4–49.9 months) from the RT of the bone metastases. For each patient, not every bone metastasis was treated: RT was prescribed primarily to relieve pain or prevent pathologic fractures as well as spinal cord compression (nine patients) or in order to reduce tumour burden when ADT started at diagnosis showed stable disease or partial response (seven patients).

Bone metastases were treated with total radiation dose from 20 to 25 Gy (4–5 Gy/fraction), from 30 to 32.5 Gy (3–6 Gy/fraction), from 54.6 to 59.8 Gy (2.1–2.3 Gy/fraction), respectively, in 7, 5 and 2 patients with IMRT technique. Two patients received SBRT with a total dose from 20 to 24 Gy (10–12 Gy/fr) on the metastases site.

### ADT administration

ADT was administrated as follows: for all patients, ADT started at the diagnosis of synchronous metastases. Seven patients received continuous ADT while 13 patients received intermittent ADT as clinical strategy. Two patients suspended ADT before RT until clinical progression.

### Follow-up procedure and definition of progression

Clinical assessment and PSA measurements were performed every three months for at least one year. PSA relapse is defined according to the Consensus Statement of the American Society of Radiation Oncology (ASTRO) as elevation of PSA level nadir + 2 ng/ml and confirmed by one measurement. Biochemical disease-free survival (b-DFS) will be measured as the time from the start date of ADT to the date of PSA elevation.

Clinical progression was defined as documented local progression, or lympho-nodular or systemic disease from the start date of ADT divided into local recurrence (in this case, the primary tumour) and distant progression, outside the RT field.

Time to castration resistant PCa was calculated from the initiation of ADT until confirmed biochemical progression in the presence of castrate serum testosterone levels (50 ng/dl or less).

OS was defined as the time from the start date of ADT to the time of death from any cause.

Gastrointestinal (GI) and genitourinary (GU) toxicity was assessed and scored according to the Radiation Therapy Oncology Group/European Organization for Research and Treatment of Cancer (RTOG/EORTC) criteria [14].

## Results

We identified 20 patients with oligometastatic PCa (cM1b) at diagnosis, who underwent external-beam RT of the primary tumour in our institute, between June 2008 and March 2016.

The characteristics of the 20 patients included in this study are summarised in Table 1.

The median age of patients at diagnosis was 64.1 years (range: 45.1–81.5) and ECOG performance status of all included patients was 0 or 1. The initial PSA median value was 14.6 ng/ml (range: 4.8–414 ng/ml) and median Gleason Score was 8 (7–10).

RT to the primary tumour (prostate) was performed after a median 10.3 months (range: 3.9–51.7) from the diagnosis of oligometastatic disease; results are shown in Table 2.

Biochemical failure occurred in 12 patients (60%) after a median time of 23.03 months (range: 14.5–104 months) from the starting of ADT.

Clinical progression was documented in 12 patients (60%), in all cases preceded by biochemical progression after a median time of 23.6 months (range: 15.4–106.1 months) from the starting of ADT and detected by radiological imaging.

Local recurrence on prostate was documented in two patients, in one patient also associated with distant bone metastases, after respectively 106.1 and 18.3 months from the start of ADT.

Distant progression with occurrence of bone metastases was documented in eight patients, including progression of irradiated metastases (one patient) and lymph-nodes progression (one patient). One patient showed only lymph-nodes progression and one only progression on RT-treated metastases.

Three patients have become castration resistant, starting a new therapy (chemotherapy or abiraterone). The median time to castration resistance was 31.03 months (range: 29.9–31.5 months).

The median follow-up from the end of radiation treatment was 26.9 months (range: 10.3–55.5), two-year OS was of 100%.

At the time of the assessment (June 2017), 18 patients (90%) are alive, among them seven are alive with evidence of disease and two died, one of PCa-related disease and one patient of secondary malignancy (glioblastoma).

RT of the primary tumour was well tolerated as shown in Table 3. According to RTOG/EORTC criteria [14], only one patient developed acute grade 3 GU toxicity. No late grade >2 adverse events were observed during the follow-up period (Table 3).

## Discussion

In recent years, several series of mPCa patients treated with the metastases-directed approach have been published, suggesting that, in selected patients, salvage treatment directed to low-volume recurrent cancer could lead to good oncologic outcomes [15–17]. In this context, primary-tumour-directed therapy seems to be the next step in order to improve survival and enhance response to systemic treatment.

Our series, based on the retrospective data of 20 patients, demonstrates promising results in terms of outcome and toxicity of cytoreductive RT of the primary tumour in patients with oligometastatic PCa. Only two patients experienced in-field progression despite primary tumour RT after 106.1 and 18.3 months from the start of ADT and two-year OS was of 100% (median follow-up 26.9 months). The outcome of the STAMPEDE standard arm A including patients treated with only ADT may act as a benchmark for our series. Considering all cohort patients, median progression-free survival (PFS) for the cohort was 11.2 months and median OS was 42.1 months. Two-year OS was 72% for the entire cohort and 75% for patients with bone metastases [18].

The rationale of metastasis-directed treatment comes from the concept that obviously, patients with oligometastatic disease are prone to progress at the initial metastatic foci, so a decreasing tumour burden would eventually allow for an improved response to systemic therapies.

In other metastatic malignancies, such as kidney, breast and ovarian cancer, the reduction of primary tumour has been proved to be associated with improvement in outcome [19]. For example, in kidney cancer, cytoreductive nephrectomy has shown a significant improvement in survival and provided the proof that uncontrolled local malignancy could promote progression or appearance of metastases [20].

Moreover, our approach was not limited to the primary tumour but included RT, in select cases, synchronous treatment of bone metastases. From the biological point of view, the relationship between the primary tumour and distant metastases is complex. What appears clear is that the primary tumour is a continuous source of new metastases with its capability of promoting tumour progression. The primary site seems to be able to release tumour cells into the circulation, enriching itself (self-seeding) and promoting distant metastases [21].

ADT alone appears not to be enough to limit tumour progression. Moreover, a growing body of studies shows that ADT could also select resistant cells with a more aggressive tumour phenotype [22, 23]. All these hypotheses could justify the addition of RT of the primary and bone metastases to ADT.

The role of local treatments (both RT and radical prostatectomy) in the metastatic setting is still under investigation in different clinical trials. Reported and already published clinical data are often from retrospective studies characterised by heterogeneous results. Although there is a paucity and diversity of published data, some studies and reviews report a potential and beneficial impact of local treatment (whatever it was) on primary tumour in mPCa [24–27].

Whether to treat the primary tumour or not in patients with mPCa has become more relevant and is a source of recent debate; hopefully, unsolved questions will be answered in the coming years by ongoing phase III trials, whose results are eagerly awaited (STAMPEDE, HORRAD, PEACE1 and NCT01751438)(Table 4).

A Multi-Stage Multi-Arm Randomised Controlled Trial STAMPEDE is the largest therapeutic randomised controlled trial in PCa. STAMPEDE trial’s control arm included men with mPCa at diagnosis receiving SOC therapy (ADT) [26].

In the light of emerging data on the role of the RT to the primary tumour in the patients with mPCa, in 2013, a new arm was added to the STAMPEDE trial in order to investigate primary RT in patients with mPCa who have at least six months of ADT before their RT (arm H) [5]. This arm will be compared to the control arm receiving ADT alone (arm A).

The Dutch randomised phase III study HORRAD started in 2004 and allocated men in stage M1b (bone) PCa with characteristics similar to our patients to receive ADT alone versus ADT plus local RT [28].

Another prospective randomised study which is currently recruiting participants is PEACE1. This multicentre phase III trial includes four arms: ADT alone, ADT + RT, ADT + Abiraterone Acetate + Prednisone, ADT + Abiraterone Acetate + Prednisone + RT [29].

A multicentre North American trial (NCT01751438) is randomising patients with mPCa to SOC (ADT or bilateral orchiectomy) alone or SOC associated to definitive local therapy (prostate RT or surgery) in mPCa patients. The primary outcome is PFS and initial results are expected in March 2018 [30].

Concerning the already published results, in a Korean study (2016), 140 patients with mPCa (M1b or M1c) 38 were treated with local RT to the primary tumour together with systemic therapy. The three-year OS rate was higher in men receiving RT compared to the other group, 69% versus 43%. The same goes for biochemical failure-free survival results, which were 52% versus 16% [31].

In another study, where more than 500 men affected by mPCa from the National Cancer Database (NCDB) received RT plus ADT, Rusthoven et al [32] demonstrated superior median (55 versus 37 months) and five-year OS (49% versus 33%) with prostate RT plus ADT compared with ADT alone.

A reduction in primary tumour with cytoreductive intent could be achieved not only with RT, but also with surgical prostatectomy which has been proposed with the same intent [17].

A recent Surveillance Epidemiology and End Results (SEER) Program population-based analysis suggested that local treatment of the prostate in mPCa patients may improve survival compared to patients without local treatment [33]. In this study, five-year OS was significantly higher in patients undergoing either radical prostatectomy (67.4%) or brachytherapy (52.6%), compared with men who did not receive any local therapy (22.5%) (p < 0.001). Considering only patients in stage M1b, five-year OS was 70.1%, 55% and 22.9% in men who underwent surgery, brachytherapy or no local treatment, respectively. Interestingly, the NCDB secondary analysis comparing the survival outcomes for patients treated with local RT plus ADT versus prostatectomy plus ADT demonstrated no significant differences in OS, whereas both therapies were superior to ADT alone [28].

Whether we talk about RT or surgery, a primary tumour local treatment associated with a systemic therapy (ADT) might be a reasonable treatment option in a selected subgroup of patients with a diagnosis of PCa with minimal osseous metastases. RT might have the advantage of a noninvasive approach and a very favourable toxicity profile; however, full evaluation of its role requires prospective controlled data.

## Conclusion

It should be noted that the heterogeneous nature of our study population strongly limited its generalisability. In addition, the small number of patients treated with local therapies and possible selection biases could affect our clinical findings.

We are aware of the several limitations of a single centre retrospective analysis. Nevertheless, our report has shown that the addition of RT to ADT is a well-tolerated treatment with a potential beneficial effect in terms of clinical outcome.

Even if supported by a strong rationale, local cytoreductive RT of the primary tumour in mPCa requires further investigation in prospective randomised clinical trials.

A careful selection of candidates is strictly necessary to identify patients that could really benefit most from this combined approach.

## Conflict of interest

The authors declare that they have no actual or potential conflicts of interest.

## Informed consent

Informed consent was obtained from all individual participants included in the study.

## Figures and Tables

**Table 1.** Patient, tumour, treatment-related variables.

Characteristics	Value
Number of patients	20
Median age at time of diagnosis (range), years	64.1 (45.1–81.5)
Median initial PSA (range), ng/ml	17 (4.8–414)
Median Gleason Score at biopsy (range)	8 (7–10)
Number of bone metastases at diagnosis, patients 1 2 3 4	11 5 3 1
Median time interval between PCa diagnosis and RT to the prostate (range), months	10.3 (3.9–51.7)
Time interval between RT to the metastases and RT to the prostate (range), months	11.6 (2.4–49.9)
RT to metastases and RT to prostate, patients Concomitant RT primary after RT metastases No RT on metastases	4 12 4
Type of ADT during RT, patients LHRH analogue alone Bicalutamide TAB	14 1 5

Legend: RT–radiotherapy; ADT–androgen deprivation therapy; LHRH–LH-Releasing hormone; TAB–total androgen blockade.

**Table 2.** Results.

Characteristics	Value
Median follow-up (range), months	26.9 months (10.3–55.5)
Biochemical failure to the RT to the primary, patients Yes No	12 (60%) 8 (40%)
Median time to biochemical failure from ADT start (range), months	23.03 months (14.5–104)
Clinical failure to the RT to the primary, patients Yes No	12 (60%) 8 (40%)
Median time to clinical failure from ADT start (range), months	23.6 months (15.4–106.1)
Developed castration resistance, patients Yes No	3 (33%) 6 (67%)
Median time to castration resistance from RT (range), months	31.03 (29.9–31.5)
Two-year OS	100%

Legend: RT–radiotherapy; ADT–androgen deprivation therapy; OS–overall survival

**Table 3.** Acute and late toxicity.

Acute RT toxicity (RTOG/EORTC criteria) [11]	Number of patients
GI	
Grade 0	15
Grade 1	3
Grade 2	2
GU	
Grade 0	9
Grade 1	9
Grade 2	1
Grade 3	1
**Late RT toxicity (RTOG/EORTC criteria)** [11]	
GI	
Grade 0	20
GU	
Grade 0	15
Grade 1	2
Grade 2	3

Legend: RT–radiotherapy; RTOG/EORTC–Radiation Therapy Oncology Group/European Organization for Research and Treatment of Cancer

**Table 4.** Ongoing trials.

Study name	Country Primary investigator	Phase	Design	Primary endpoint	Start	Accrual	NCT/NRT number
**Stampede** [16]	UK, N D James, MD University Hospital Birmingham	Phase II and Phase III	SOC (ADT) and prostate RT, (two possible RT schedules)	OS	January 2013 (Arm H)	Close to recruitment in September 2016 (Arm H)	NCT00268476
**Horrad** [24]	Netherlands, G van Andel, MD Onze Lieve Vrouwe Gasthuis, Amsterdam	Phase II	SOC (ADT) vs SOC (ADT) and prostate RT	OS	June 2004	Close to recruitment in August 2014	NRT 271 www.trialregister.nl
**PEACE 1** [25]	Europe, K Fizazi, MD PhD Gustave Roussy, Cancer Campus Grand Paris, Paris	Phase III	SOC (ADT +/- Docetaxel) +/- abiraterone acetate with or without prostate RT	OS, PFS	October 2013	May 2017 (Final data collection)	NCT01957436
**BST +/- definitive treatment** [26]	USA, B F Chapin, MD M.D. Anderson Cancer Center, Houston	Phase II	SOC (ADT or BO) vs SOC (ADT or BO) with or without surgery or prostate RT	PFS	December 2012	March 2018 (Final data collection)	NCT01751438
